# Number of people requiring post-exposure prophylaxis to end leprosy: A modeling study

**DOI:** 10.1371/journal.pntd.0009146

**Published:** 2021-02-25

**Authors:** Anneke T. Taal, David J. Blok, Wim H. van Brakel, Sake J. de Vlas, Jan Hendrik Richardus

**Affiliations:** 1 NLR, Amsterdam, The Netherlands; 2 Department of Public Health, Erasmus MC, University Medical Center Rotterdam, Rotterdam, The Netherlands; Institute of Continuing Medical Education of Ioannina, GREECE

## Abstract

**Background:**

Worldwide, around 210,000 new cases of leprosy are detected annually. To end leprosy, i.e. zero new leprosy cases, preventive interventions such as contact tracing and post-exposure prophylaxis (PEP) are required. This study aims to estimate the number of people requiring PEP to reduce leprosy new case detection (NCD) at national and global level by 50% and 90%.

**Methodology/Principal findings:**

The individual-based model SIMCOLEP was fitted to seven leprosy settings defined by NCD and MB proportion. Using data of all 110 countries with known leprosy patients in 2016, we assigned each country to one of these settings. We predicted the impact of administering PEP to about 25 contacts of leprosy patients on the annual NCD for 25 years and estimated the number of contacts requiring PEP per country for each year. The NCD trends show an increase in NCD in the first year (i.e. backlog cases) followed by a significant decrease thereafter. A reduction of 50% and 90% of new cases would be achieved in most countries in 5 and 22 years if 20.6 and 40.2 million people are treated with PEP over that period, respectively. For India, Brazil, and Indonesia together, a total of 32.9 million people requiring PEP to achieve a 90% reduction in 22 years.

**Conclusion/Significance:**

The leprosy problem is far greater than the 210,000 new cases reported annually. Our model estimates of the number of people requiring PEP to achieve significant reduction of new leprosy cases can be used by policymakers and program managers to develop long-term strategies to end leprosy.

## Author summary

Leprosy is a chronic infectious disease affecting the skin and peripheral nerves. Despite efforts to end leprosy, the number of new leprosy cases remains stable at around 210,000. To interrupt transmission and reduce this number significantly, new strategies such as the provision of (prophylactic) treatment need to be implemented. This should be done by targeting those most at risk of developing leprosy, the contacts of new leprosy patients. Mathematical modeling can be applied to predict the impact of preventive treatment with post-exposure prophylaxis (PEP) on the incidence and the number of contacts requiring PEP at global and national level. In this study, the cumulative number of individuals requiring PEP was estimated at 40.2 million globally to reduce the incidence by 90% in 22 years. This number provides a more realistic reflection of the size of the leprosy problem worldwide. The estimates at national level are highly relevant to all countries where leprosy has been reported as it can support the policymakers and health program makers to design their elimination strategies. It also provides insight into the investment that is required to end leprosy globally and nationally.

## Introduction

Leprosy is a chronic infectious disease that can affect the skin and peripheral nerves. Untreated, it may lead to physical and mental disabilities. Leprosy is treated with multidrug therapy (MDT), a combination of antibiotics including rifampicin, dapsone, and clofazimine [1]. Transmission of *Mycobacterium leprae*, the causative agent of leprosy, is mainly human-to-human via the respiratory route. Once infected, it can take two to twelve years before the first signs and symptoms appear, although only a small proportion will develop the disease [2]. Worldwide, 208,619 new cases of leprosy were detected and reported in 2018, of which 80% in India, Brazil, and Indonesia [3].

Elimination of leprosy has been a goal for many years. In 1991 published WHO set a target of less than one registered case per 10,000 population by 2000. Even though this elimination target was met at global level, high case detection numbers persisted at subnational level in high endemic countries [4]. From 2000 onwards, the same target was set to be reached at country-level by 2005 and ‘elimination as a public health problem’ was achieved in many countries. The national efforts to reach elimination was also reflected in a steep decline in the new cases detection (NCD) up to 2005 [5]. Smith *et al*. (2015) however, argued that this steep decline was primarily a result of the reduction in leprosy control activities seen after elimination was declared in 2000 [6]. From 2005 onwards, the NCD has remained stable at around 210,000 cases with high proportions of disability grade 2 (DG2) and child cases among the new cases, indicating detection delay and continuing transmission respectively [3].

For many decades, leprosy control has been based on (passive) case detection and provision of MDT. Meanwhile, new preventive interventions have been developed, such as contact tracing combined with the provision of post-exposure prophylaxis (PEP) with single-dose rifampicin (SDR). The COLEP trial in Bangladesh has shown that this combination may reduce the risk of developing leprosy in contacts by 57%. SDR-PEP, therefore, is a promising new leprosy elimination strategy [1, 7]. To interrupt transmission of *M*. *leprae* and reduce the NCD significantly these combined interventions need to be implemented in the most effective way, by targeting those most at risk of developing leprosy.

Estimating the number of people requiring PEP at global and national level could support policymakers and investors to develop effective elimination strategies. As described by Cunha *et al*. (2015), in six small scale PEP studies, the number of people requiring treatment with PEP to prevent one case has been estimated to vary between 14 and 265 [8]. These estimates however, do not account for the potential reduction in NCD over a longer period of time due to PEP. It remains unknown how long it will take to achieve a substantial reduction in NCD using PEP and what the total number of people requiring PEP would be at country and global level.

As the long-term impact of PEP on NCD, and the associated number of people requiring PEP cannot be established through clinical trials or short-term observational studies, we apply mathematical modeling. This is an efficient and powerful tool for quantifying transmission patterns, predicting future trends in leprosy detection and assessing the potential long-term impact of interventions [9]. Mathematical modeling has been applied before to predict the course of leprosy trends, and to assess the feasibility of elimination and the impact of interventions, including contact tracing and PEP, in various settings [10–12]. In this study, we use the established individual-based model SIMCOLEP to estimate the number of people requiring PEP to reduce leprosy new case detection at national and global level by 50% and 90%.

## Methods

The stochastic individual-based model SIMCOLEP simulates the spread of *M*. *leprae* in a population that is structured in households and of the impact of treatment and interventions, including administering SDR to contacts. A full description of the model can be found in Fischer *et al*. (2010) [13] and Blok *et al*. (2015) [14]. The model simulates life-histories of individuals, including birth, death, and formation of households. During the simulation individuals can change households due to life events such as marriage, moving after adolescence, becoming a widow(er) and death.

In the model, transmission of *M*. *leprae* occurs when a susceptible individual has contact with an infectious individual. We assumed that 20% of the population is susceptible and that 80% will not develop leprosy. The transmission is modeled through two separate transmission processes: 1) transmission in the general population and 2) within households. The latter reflects the increased risk of acquiring the infection if one or more household contacts are infected. Infectivity is determined by the product of the contact rate and the probability of infection during a contact. The natural history of infection is modeled as presented in Meima *et al*. (1999) [15]. An infected individual develops either paucibacillary (PB) or multibacillary (MB) leprosy, which is randomly assigned based on the observed MB proportion. We assumed that only MB leprosy is infectious. After infection, an individual enters the asymptomatic state, which on average lasts 4.2 years for PB and 11.1 years for MB [13, 16]. Afterwards the individual proceeds to the symptomatic state. A PB case may self-heal, while an MB case remains symptomatic until treatment or death. Table A in Supporting Information File 1 provides an overview of all leprosy transmission parameters.

SIMCOLEP also replicates control measures including treatment with MDT, passive case detection, tracing, and screening of contacts of an index patient and the provision of PEP. Only patients with clinical leprosy can be diagnosed. All diagnosed patients receive MDT treatment and are assumed not to be infectious anymore after treatment. A cured patient can relapse with a rate of 0.001 per year: 90% relapses to MB and 10% to PB [17]. We further included the protective effect of BCG vaccination prior to infection, which is set to 60% [18]. (Table A in Supporting Information File 1).

### Modeled leprosy settings

The model was calibrated to the demographic and leprosy situation of seven well-known settings: India, Brazil, and Indonesia at country-level, and Chhattisgarh (India), Pará (Brazil), Madura (Indonesia), and Nilphamari and Rangpur (Bangladesh). This study used the previously published model calibrations [11, 13].

First, we calibrated household formation parameters to match the observed household distribution in each modeled setting. Table B and C in Supporting Information File 1 provide an overview of the data used and calibrated parameters. Second, we quantified the model to the leprosy situation in the seven settings [11, 13]. Data used to fit leprosy trends include the reported new case detection rate (NCDR) and the observed MB proportion (Table D in Supporting Information File 1). The MB proportion was 50%, 65%, 80% and 20% in India, Brazil, Indonesia, and Bangladesh, respectively. We also modeled leprosy control programs in each setting. The quality of the program was reflected by passive detection delays, which were on average two years in India, Indonesia, and Bangladesh, and three years in Brazil. The coverage of existing household contact tracing was set to 0%, 59%, 11%, and 90% in India, Brazil, Indonesia, and Bangladesh, respectively, prior to the administration of post-exposure prophylaxis (Table A in Supporting Information File 1).

The transmission contact rates in the general population were calibrated to match the observed NCDR in each modeled setting. The NCDR (mean of 100 runs) were compared to the data using a log-likelihood function assuming a Poisson distribution. Afterwards, we used a polynomial regression, in which we fitted the obtained likelihood ratios, as a metamodel to derive the optimal value of the contact rates (Table A in Supporting Information File 1). For India, Brazil and Indonesia, validation was performed by assessing the ability to predict the NCDR in 2015. A detailed description of the fitting procedure can also be found elsewhere [10, 11].

### Post-exposure prophylaxis (PEP)

For each setting, we assessed the impact of intensive contact screening and the provision of PEP to contacts of an index patient. In all settings, we increased the coverage of contact tracing and screening to 90% when PEP was introduced. Besides the household members of the index patient, four additional neighboring households were traced and screened for leprosy. In the model, these households are selected randomly at the time of screening. As a result, on average 20–25 persons per index patient are traced and screened for leprosy depending on the household size of the setting (Table A in Supporting Information File 1). This number was chosen because the Leprosy Post-Exposure Prophylaxis program has demonstrated that this is a feasible strategy [19]. In the model, eligible contacts without any signs of clinical leprosy are given PEP, while those with signs of clinical leprosy are given MDT. The effectiveness of PEP (based on the experience with SDR-PEP) was set to 50% among household contacts and 70% among other (non-household) contacts [7]. The compliance of eligible contacts was set to 99% [19]. Predictions of the impact of PEP on the number of new cases per 100,000 and the number of individuals requiring PEP per 100,000 were generated for a period of 25 years. Fig A and Fig B in the Supporting Information File 1 show the modeled NCDR and individual required PEP for each setting, respectively.

### Estimation of population requiring PEP

Leprosy and population data of India, Brazil and Indonesia have been collected from national health systems (sources) and of the remaining 107 countries that reported leprosy cases in 2016 from the WHO’s Weekly Epidemiological Record [20]. To estimate the population requiring PEP in each country, we categorized each country to one of the modeled settings presented in Table 1. As India, Brazil, and Indonesia have the largest number of cases globally, we categorized these countries at a state-level. Each country or state was matched to a setting that would have the best fit in terms of number of cases, NCDR and MB/PB rate. Table E in Supporting Information File 1 provides an overview of the categorization.

**Table 1:** Overview of the main characteristics of the modelled seven leprosy settings, including the new case detection rate (NCDR), multibacillary (MB) and paucibacillary (PB) ratio and the household size.

Setting	NCDR	MB/PB ratio	Household size (average)	Model quantification based on
A	30/100 000	50/50	4.9	Chhattisgarh, India^a^
B	40/100 000	65/35	4.5	Para State, Brazil^a^
C	45/100 000	80/20	4.0	Madura, Indonesia^a^
D	25/100 000	20/80	4.6	Nilphamari and Rangpur, Bangladesh^b^
E	10/100 000	50/50	4.9	India country-level^a^
F	10/100 000	65/35	4.5	Brazil country-level^a^
G	5/100 000	80/20	4.0	Indonesia country-level^a^

a Model quantification published in Blok, De Vlas and Richardus (2015) [14]

b Model quantification published in Fischer *et al*. (2010) [13]

The outcome measure for this study is NCD. For each country, we projected the future trend of new cases based on the number of new cases in 2016 and the modeled NCDR trend of the matched setting. We multiplied the population size of a country with the modeled NCDR to calculate the NCD for each year (Equation A in Supporting Information File 1) The number of individuals that required to receive PEP per year was calculated in the same way.

Results are shown by country and summarized for three groups: 1) three most endemic countries India, Brazil and Indonesia; 2) 19 countries that were also declared to be priority countries by the WHO; and 3) remaining 88 countries that reported indigenous new cases of leprosy in 2016 [10].

### Sensitivity analysis

We performed a sensitivity analysis on the effectiveness of PEP and the infectiousness of PB. First, we assessed to what extent lowering the effectiveness of PEP in other contacts to 50% would affect the number of people requiring PEP and the reduction in NCD. Second, as PB leprosy was assumed not to be infectious in our main results, we also assessed how outcomes would change if we would assume that it would be infectious. Since it is unknown to what extent PB leprosy is infectious, we tested our model assuming a PB infectivity of 0.1 and 0.2.

## Results

**Fig 1:**
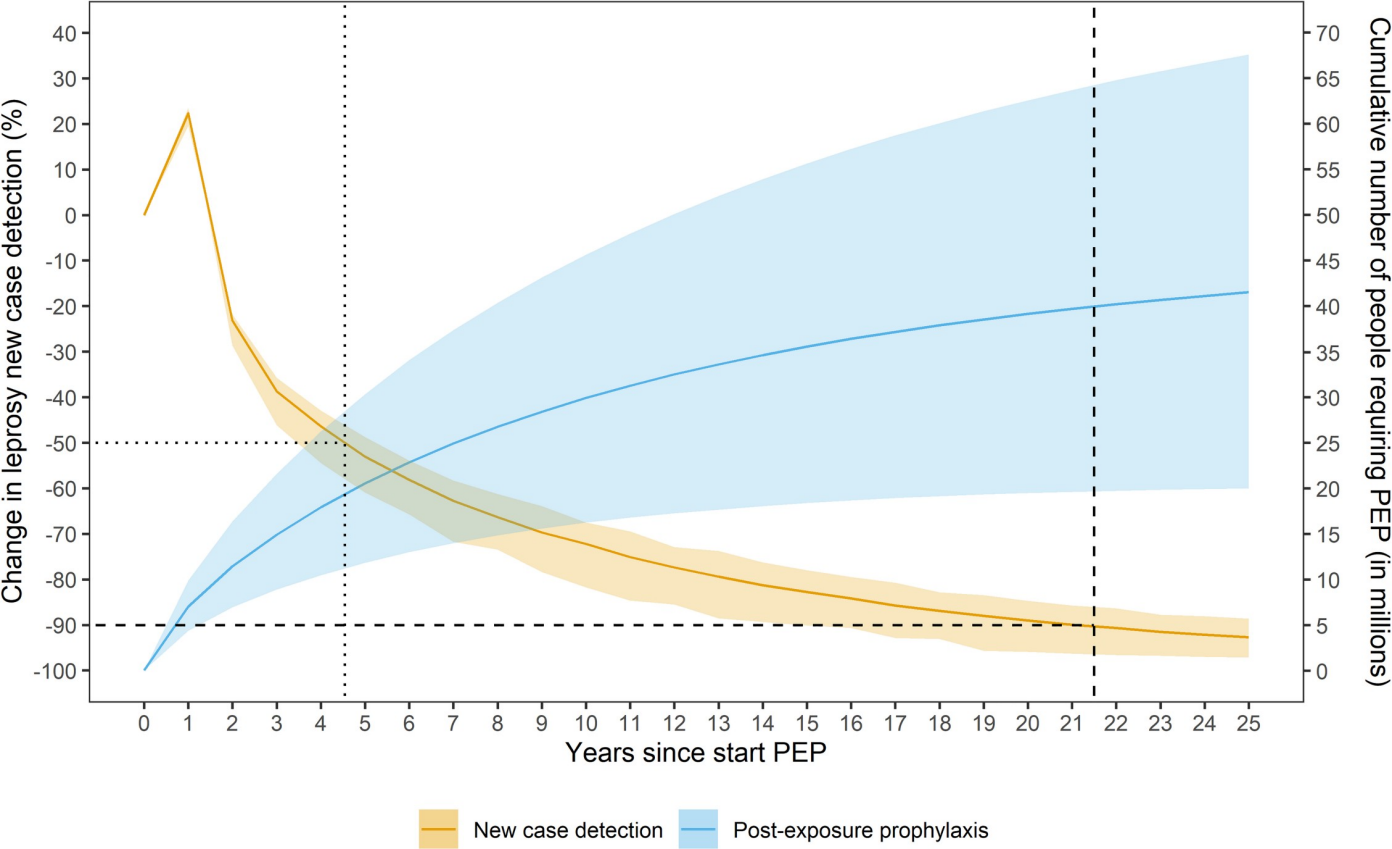
The global change in new case detection and cumulative number of people requiring post-exposure prophylaxis (PEP) over a period of 25 years. The orange line represents the change in the new case detection (NCD) and its 95% confidence interval. The blue line is the cumulative number of people requiring post-exposure prophylaxis and its 95% confidence interval. The dotted and dashed vertical line represents the time of 50% and 90% reduction in NCD, respectively.

Fig 1 and Table 2 show the global change in the new case detection (NCD) and the cumulative number of people that requires PEP when given to the household of the index patient and four neighboring households (i.e. 20–25 contacts). In 2016 (t = 0), 228,970 new cases were reported to the WHO by 110 countries. When starting with offering PEP to contacts, the NCD trend shows an increase of 20% in the NCD in the first year due to backlog cases, immediately followed by a significant decline that flattens towards the end (t = 25). A 50% and 90% reduction in NCD could be reached in about 5 and 22 years, respectively. The model predictions show that a total of 20.6 (95% CI: 11.9–29.9) million people requiring PEP to achieve a 50% reduction and an additional 19.6 (total of 40.2; 95% CI: 19.8–64.8) million people to achieve a 90% reduction.

**Table 2:** Number of people requiring post-exposure prophylaxis (PEP) to reduce the new case detection (NCD) by 50% and by 90% at global level and in the three country groups.

		50% reduction					90% reduction				
	NCD at time 0	Duration (years)	NCD		People requiring PEP (million)		Duration (years)	NCD		People requiring PEP (million)	
			Mean	Range^a^	Mean	Range^a^		Mean	Range^a^	Mean	Range^a^
**Global**	228,970	5	107,520	48,970–152,290	20.6	11.9–29.9	22	21,400	4,800–44,690	40.2	19.8–64.8
**Group 1**^**b**^	188,430	5	86,780	47,360–131,970	16.8	10.3–24.3	22	17,770	4,570–36,180	32.9	17.3–52.0
**Group 2**^**c**^	30,450	6	13,680	4,730–24,070	3.2	1.3–5.1	21	3,030	260–7,080	5.4	1.9–9.5
**Group 3**^**d**^	10,080	6	4,520	1,470–8,080	1.0	0.4–1.7	21	990	60–2,310	1.8	0.6–3.1

a 95% confidence interval reflecting the stochastic variation of 1,000 runs

b India, Brazil, and Indonesia

c DR Congo, Ethiopia, Nepal, Bangladesh, Myanmar, Tanzania, Sri Lanka, Madagascar, Philippines, Nigeria, Mozambique, Ivory Coast, South Sudan, Egypt, Sudan, Angola, Comoros, Kiribati, Micronesia

d 88 remaining countries

For Group 1 (India, Brazil, and Indonesia), a total of 16.8 million people requiring PEP to achieve a 50% reduction in on average 5 years (Table 2). For Group 2 (19 other WHO priority countries) and Group 3 (remaining 88 countries) this is 3.2 and 1.0 million people in on average 6 years, respectively. To achieve a 90% reduction in NCD for Group 1, a total of 32.9 million people requiring PEP in on average 22 years. For Group 2 and 3, this is 5.4 and 1.8 million people in on average 21 years, respectively.

Table 3 shows the number of people requiring PEP to achieve a 50% and 90% reduction in NCD for all WHO priority countries (i.e. Group 1 and 2) separately. For India, Brazil, and Indonesia, 22.3, 7.4 and 3.2 million people requiring PEP to achieve a 90% reduction in 22, 20 and 25 years, respectively. For Democratic Republic of Congo, Ethiopia, and Bangladesh this is between 640,000 and 750,000 people in 19, 22, and 23 years, respectively. For 13 countries, it is between 100,000 and 450,000 people, and for Comoros, Micronesia, and Kiribati it is below 60,000 people in 22, 22, and 23 years, respectively.

**Table 3:** Number of people requiring post-exposure prophylaxis (PEP) per country to reduce the new case detection (NCD) by 50% and by 90% in the 22 WHO priority countries, ordered according to the NCD.

				50% reduction					90% reduction				
	Country	Population at time 0 (million)	NCD at time 0	Duration (years)	NCD		People requiring PEP (thousands)		Duration (years)	NCD		People requiring PEP (thousands)	
					Mean	Range^a^	Mean	Range^a^		Mean	Range^a^	Mean	Range^a^
**GROUP 1**	India	1,324.2	135,485	4	67,450	35,570–104,700	9,920	5,550–14,900	22	12,850	2,000–28,490	22,270	9,710–37,490
	Brazil	207.7	36,093	6	15,960	11,630–21,520	4,410	3,290–5,550	20	3,540	1,970–5,570	7,420	5,250–9,890
	Indonesia	261.1	16,826	7	7,760	5,460–10,230	1,830	1,370–2,290	25	1,580	9,10–2,690	3,180	2,210–4,140
**GROUP 2**	DR Congo	78.7	3,742	5	1,810	990–2,730	410	240–600	19	350	90–670	750	400–1,160
	Ethiopia	102.4	3,692	6	1,730	470–3,040	360	100–610	22	360	0–840	640	160–1,160
	Nepal	29.0	3,054	4	1,450	380–2,850	220	80–380	19	280	0–890	450	110–900
	Bangladesh	163.0	3,000	7	1,400	870–1,970	450	310–600	23	290	100–510	710	445–1,020
	Myanmar	528.9	2,609	6	1,220	330–2,150	260	70–430	22	250	0–590	450	110–820
	Tanzania	55.6	2,047	6	960	260–1,670	200	60–340	22	200	0–470	360	90–650
	Sri Lanka	21.2	1,977	4	940	250–1,840	150	60–250	19	197	0–580	290	70–590
	Madagascar	24.9	1,780	6	840	220–1,470	180	50–290	22	170	0–410	310	80–560
	Philippines	103.3	1,721	6	810	220–1,420	170	50–280	22	170	0–390	300	80–540
	Nigeria	186.0	1,362	6	640	170–1,130	140	40–250	22	130	0–310	240	60–430
	Mozambique	28.8	1,289	6	610	160–1,070	130	40–220	22	120	0–290	230	60–410
	Ivory Coast	23.7	895	5	430	240–650	100	60–150	19	85	20–160	180	100–280
	South Sudan	12.2	691	6	330	90–570	70	20–120	22	65	0–160	120	30–220
	Egypt	95.7	651	6	310	80–540	70	20–110	22	60	0–150	120	30–210
	Sudan	39.6	624	6	290	80–520	60	20–110	22	60	0–140	110	30–200
	Angola	28.8	619	6	290	80–510	60	20–110	22	60	0–140	110	30–200
	Comoros	0.8	310	4	150	80–230	30	10–40	22	30	10–70	60	30–90
	Kiribati	0.1	218	7	100	60–140	40	20–50	23	20	10–40	60	40–80
	Micronesia	1.0	169	4	80	50–130	20	10–30	22	10	0–40	30	20–50

a 95% confidence interval reflecting the stochastic variation of 1,000 runs

The number of people requiring PEP for high and low endemic provinces/states in India, Brazil and Indonesia are shown in Table 4. The duration until a 50% reduction will be achieved in high and low endemic states is different in India, Brazil, and Indonesia. In India, a 90% reduction will be achieved in high endemic states in 22 years and in low endemic states in 20 years. In Brazil, this is in 21 years and 19 years in high and low endemic states, respectively. Indonesia will achieve a 90% reduction in 25 years in high endemic provinces and in 22 years in low endemic provinces.

**Table 4:** Number of people requiring post-exposure prophylaxis (PEP) to reduce the new case detection (NCD) by 50% and by 90% in high and low-endemic states in India, Brazil and Indonesia.

			50% reduction					90% reduction				
Country		NCD at time 0	Duration (years)	NCD		People requiring PEP (million)		Duration (years)	NCD		People requiring PEP (million)	
				Mean	Range^a^	Mean	Range^a^		Mean	Range^a^	Mean	Range^a^
India	High	107,097	5	47,660	23,650–73,580	9.1	5.3–13.4	22	10,700	2,000–21,930	18.0	8.8–29.1
	Low	28,388	4	13,520	3,530–26,480	2.0	0.7–3.6	20	2,490	0–6,810	4.3	1.0–8.5
Brazil	High	28,749	6	12,950	9,910–16,480	4.1	3.3–5.0	21	2,710	1,550–3,920	7.9	5.8–10.0
	Low	7,344	5	3,650	2,010–5,540	1.6	0.9–2.3	19	720	180–1,370	3.3	1.7–5.1
Indonesia	High	14,642	7	6,820	5,260–8,500	1.6	1.3–1.9	25	1,420	840–2,040	2.8	2.1–3.5
	Low	2,214	6	1,040	280–1,900	0.2	0.1–0.4	22	210	0–510	0.4	0.1–0.7

a 95% confidence interval reflecting the stochastic variation of 1,000 runs

Table 5 shows that lowering the effectiveness of PEP would not result in a substantially higher NCD and higher number of people requiring PEP as compared to the main results. When assuming that PB is infectious, the PEP intervention would result in a slightly lower NCD and number of people requiring PEP.

**Table 5:** Sensitivity analysis.

	New case detection (proportion)						Cumulative number of people requiring PEP (proportion)				
	0yrs ^a^	5yrs ^a^	10yrs ^a^	15yrs ^a^	20yrs ^a^	25yrs ^a^	1-5yrs^a^	1-10yrs^a^	1-15yrs^a^	1-20yrs^a^	1-25yrs^a^
**Main results** ^**b**^	280,066	107,520	63,550	39,490	25,150	16,750	20,548,000	29,926,000	35,554,000	39,145,000	41,520,000
**SDR effectiveness 50% for all contacts**	0	+200 (+0.2%)	+130 (+0.2%)	-100 (-0.3%)	+240 (+0.1%)	+100 (+0.6%)	+15,000 (+0.1%)	+43,000 (+0.1%)	+83,000 (+0.2%)	+103,000 (+0.3%)	+111,000 (+0.3%)
**PB infectivity 0.1**	0	-280 (-0.3%)	-2,170 (-3.4%)	-1190 (-4.2%)	-240 (-1.0%)	-140 (-0.8%)	-199,000 (-1.0%)	-396,000 (-1.3%)	-510,000 (-1.4%)	-584,000 (-1.5%)	-611,000 (-1.5%)
**PB infectivity 0.2**	0	-910 (-0.8%)	-2,640 (-4.2%)	-1,640 (-4.2%)	-600 (-2.4%)	-620 (-3.7%)	-144,000 (-0.7%)	-455,000 (-1.5%)	-646,000 (-1.8%)	-797,000 (-2.0%)	-886,000 (-2.1%)

a Years since start PEP

b Assumptions: SDR effectiveness 50% in household contacts and 70% in other contacts; PB not infectious

## Discussion

The NCD trends show an increase in NCD in the first year (i.e. backlog cases) followed by a significant decrease thereafter. We predicted that a reduction of 50% and 90% of new cases could be achieved in most countries in 5 and 22 years if 20.6 and 40.2 million people are treated with PEP over that period, respectively. For India, Brazil, and Indonesia together, a total of 32.9 million people would require PEP to achieve a 90% reduction in 22 years.

There are marked differences in the years needed to achieve a 50% or 90% reduction among the countries in the three groups due to differences in epidemiologic and demographic factors, i.e. MB proportion and household size. For example, the model predicted that India, Nepal, Sri Lanka, Comoros, and Micronesia (all modeled to setting A or E, see Table 1) will achieve a 50% reduction in 4 years compared to Indonesia, Bangladesh, and Kiribati (all modeled to setting D or G, see Table 1) in 7 years. Countries with a high MB proportion (e.g. Indonesia 80% MB) will take longer to achieve a reduction in NCD than in countries with a lower MB proportion (e.g. Nepal 50% MB). It is known that untreated MB patients often have a higher bacterial load and are more likely to transmit the bacteria to others compared to PB patients [21]. Moreover, the time between infection and clinical disease is longer in MB compared to PB leprosy [15]. Therefore, it is likely that in countries with a higher MB proportion a higher level of transmission takes place over a longer period, resulting in a longer time before a 90% reduction is achieved compared to countries with a low MB proportion. Also, there are differences in the duration at subnational level in India, Brazil, and Indonesia. Table 4 shows that it takes longer to achieve a 50% or 90% reduction in high endemic states compared to low endemic states.

It is promising that a 50% reduction in the global NCD could be achieved within 5 years at global level, especially for program managers who are planning their five year-strategies. At a country level it may take up to seven years. To reach a 50% reduction in global NCD within 5 years however, we assumed that PEP would be implemented in all countries and districts at the same time, which would be very challenging. It is therefore very likely that the actual time needed to achieve a 50% reduction would be longer. Once a 50% reduction in NCD has been achieved it will take on average another 17 years to achieve a 90% reduction in most countries and many more years to eventually reach zero new cases. The reason why it takes so much time is mainly because of the long incubation time where some people will continue to develop leprosy even after transmission has completely ceased, as well as the modeled PEP intervention strategy that relies on passive case detection to identify the index patient. In our model, PEP is continuously provided to on average 20–25 contacts of an index leprosy patient, and with declining numbers the reach of the intervention would be limited, which slows down the impact of the intervention. The time to reach a 90% reduction in NCD however, could be shortened if more effective strategies for early detection are implemented, such as extending the number of contacts provided with PEP, a repeat / second dose after 2 years, or the use of a diagnostic test to identify sub-clinical leprosy in combination with providing an enhanced PEP regimen. WHO has modeled such a strategy for tuberculosis and showed that by optimizing current tools and introducing new prophylactic treatment and a future vaccine, the decline in the global trend of tuberculosis would be accelerated from 2% to 17% per year [22].

The feasibility of contact tracing and PEP was explored in the Leprosy Post-Exposure Prophylaxis program (LPEP) study conducted in seven countries. The LPEP study showed that PEP is safe and could be integrated in routine leprosy control activities, and that there is a high level of acceptance by patients, contacts and health staff [23]. Moreover, the contact tracing and PEP was shown to be a cost-effective strategy in the short (5 years) and long term (25 years) [24]. The cost of contact tracing, screening and SDR-PEP was estimated to be US$ 2.9 per contact [25].

An important concern regarding the implementation of SDR-PEP is anti-microbial restistance to rifampicin. To promote resistance, there must be a large pool of bacilli and several doses of rifampicin must be given over a short time. This applies equally to *M*. *tuberculosis* and *M*. *leprae*. The risk of SDR causing rifampicin resistance in either infection is considered negligible [26]. The sporadic cases of rifampicin resistance in leprosy have developed over decades (and not become at all widespread), and are most likely due to TB treatment (which contains rifampicin but no other anti-leprosy drug) given to someone unknowingly harbouring large numbers of *M*. *leprae* [27]. Chemoprophylaxis with three months of isoniazid and rifapentine is now being recommended for TB programmes worldwide [28]. Thus, regimens containing rifampicin but no other anti-leprosy drug are being prescribed to large numbers of people, some of whom may be infected with *M*. *leprae*. Based on currently available evidence, the benefit of chemoprophylaxis in both TB and leprosy is considered much greater than the risk from future drug resistance. Important conditions are that leprosy contacts are screened for leprosy and TB before receiving SDR-PEP, have not received rifampicin in the past two years, and that drug resistance is monitored routinely in leprosy and TB control programs.

The estimation of the number of people requiring PEP to achieve a given reduction in new cases could to some extent be compared to the concept of the ‘population at risk’. The population at risk for leprosy has not been estimated yet due to major challenges such as the lack of an accepted screening test for sub-clinical infection, defined geographic risk areas and data from prevalence surveys. In other Neglected Tropical Diseases, the number of people requiring preventive intervention has been used as a proxy of the population at risk. For example, in lymphatic filariasis, the population at risk was defined as ‘the number of people to be targeted by mass drug administration’ to reach elimination estimated using mathematical modeling [29]. This definition is considered practical for program managers and pharmaceutical companies because it can help identify geographical target populations easily and operational indicators such as the number of drugs needed and associated costs. In leprosy, however, the number of people requiring PEP does not completely reflect the population at risk, because it does not refer to the total population in a geographical area, but to high risk groups. Also, the risk of acquiring infection or developing disease varies from person to person due to genetic factors. In household settings, children of leprosy affected parents are at higher risk to acquire the disease than spouses [30]. Identifying those that are genetically susceptible to the disease is difficult and would require reliable diagnostics, which are not available yet.

The quality of reported data is a concern in this study. The data used are from the patients registered at health centers and are mainly obtained from the WHO reports that rely on country reporting. Also, these data do not include information on the distribution of leprosy within countries [20]. Better (within) country-based leprosy data would produce more reliable estimates for each country individually. In addition, the reported figures are likely underestimating the true number of cases because of underdiagnoses and underreporting [31, 32]. A previous modeling study of a high-endemic state in Brazil estimated that the annual number of new cases could be twice that of the diagnosed and reported cases [32].

Another limitation is the uncertainty around some assumptions regarding leprosy transmission because of the paucity of evidence and data to quantify the model. For example, in our model’s main results, we assumed that PB leprosy is not infectious, because of the lack of evidence on the extent of infectiousness of PB leprosy. Therefore, we performed a sensitivity analysis to assess the importance of this assumption. Increasing the infectiousness of PB to 0.1 or 0.2 did not result in significant changes in the impact of PEP on NCD or number of people requiring PEP as compared to the initial assumption that PB is not infectious (Table 5). Also, environmental and socioeconomic factors are not included in the model. Leprosy mainly affects marginalized populations and the risk of developing the disease is associated with poor socioeconomic and environmental indicators [33, 34]. Finally, we did not model each country separately but rather chose to assign them to seven previously modelled settings that reflect a certain endemicity level and MB proportion. As a result, estimates for one of these countries are likely more reliable than those that were assigned to one of the settings [11]. The estimates of the remaining 106 countries can at best be considered as crude. Nevertheless, since India, Brazil, and Indonesia cover more than 80% of the new cases globally, this limitation will hardly affect the global number requiring PEP estimate significantly.

## Conclusion

The leprosy problem is far greater than the 210,000 new cases reported annually, and the estimates on the number of people requiring PEP to achieve a significant reduction in new leprosy cases over the years can be used by policymakers and program managers to develop long-term strategies to end leprosy (i.e. zero new leprosy cases).

## Supporting information

S1 Supporting Information File Text document containing 1) quantification and assumptions of leprosy transmission parameters (Table A), 2) demographic data used (Table B), 3) quantification of household movement parameters (Table C), 4) epidemiologic data used (Table D), 5) overview of countries’ characteristics (Table E), 6) predicted impact of PEP on the leprosy new case detection rate in seven settings (Fig A), 7) predicted number of people requiring PEP per 100,000 in seven leprosy settings (Fig B), and 8) method to calculate country specific number of individuals requiring PEP (Equation A).
(PDF)

## References

[1] World Health Organization. Guidelines for the Diagnosis, Treatment and Prevention of Leprosy. 1st ed. Geneva: WHO; 2018. 106 p.

[2] Bratschi MW, Steinmann P, Wickenden A, Gillis TP. Current knowledge on Mycobacterium leprae transmission: a systematic literature review. Lepr Rev. 2015;86:142–55. 26502685

[3] Global leprosy update, 2018: moving towards a leprosy free world. Wkly Epidemiol Rec. 2019; 94(35/36):389–412.

[4] World Health Organization. Leprosy—Global target attained. Wkly Epidemiol Rec. 2001;76(20):149–56. Available from: http://www.who.int/docstore/wer/pdf/2001/wer7620.pdf 11395918

[5] Global leprosy situation, 2006. Wkly Epidemiol Rec. 2006;81(32):309–16. 16903018

[6] Smith WC, Van Brakel WH, Gillis TP, Saunderson P, Richardus JH. The missing millions: a threat to the elimination of leprosy. PLoS Negl Trop Dis. 2015;9(4):e0003658. 10.1371/journal.pntd.0003658 25905706 PMC4408099

[7] Moet FJ, Pahan D, Oskam L, Richardus JH, COLEP Study Group. Effectiveness of single dose rifampicin in preventing leprosy in close contacts of patients with newly diagnosed leprosy: cluster randomised controlled trial. BMJ. 2008;336(7647):761–4. 10.1136/bmj.39500.885752.BE 18332051 PMC2287265

[8] Souza da Cunha S, Adjunto P, Bierrenbach LA, Barreto HLV. Chemoprophylaxis to control leprosy and the perspective of its implementation in Brazil: a primer for non-epidemiologists. Rev Inst Med Trop. 2015;57(6):481–7. 10.1590/S0036-46652015000600004 PMC4727133 27049701

[9] Medley GF, Blok DJ, Crump RE, Hollingsworth TD, Galvani AP, Ndeffo-Mbah ML, et al. Clinical infectious diseases policy lessons from leprosy modeling. Clin Infect Dis. 2018;66(Suppl 4):S281–285. 10.1093/cid/ciy005 29860289 PMC5982730

[10] De Matos JH, Blok DJ, De Vlas SJ, Richardus JH. Leprosy new case detection trends and the future effect of preventive interventions in Pará State, Brazil: a modelling study. PLoS Negl Trop Dis. 2016;10(3):e0004507. 10.1371/journal.pntd.0004507 26938738 PMC4777416

[11] Blok DJ, De Vlas SJ, Richardus JH. Global elimination of leprosy by 2020: are we on track? Parasit Vectors. 2015;8(548):1–9. 10.1186/s13071-015-1143-4 26490878 PMC4618543

[12] Blok DJ, Crump RE, Sundaresh R, Ndeffo-Mbah M, Galvani AP, Porco TC, et al. Forecasting the new case detection rate of leprosy in four states of Brazil: A comparison of modelling approaches. Epidemics. 2017;18:92–100. 10.1016/j.epidem.2017.01.005 28279460 PMC6198811

[13] Fischer EAJ, de Vlas SJ, Meima A, Habbema JDF, Richardus JH. Different mechanisms for heterogeneity in leprosy susceptibility can explain disease vlustering within households. PLoS One. 2010;5(11):e14061. 10.1371/journal.pone.0014061 21124916 PMC2988824

[14] Blok DJ, de Vlas SJ, Fischer EAJ, Richardus JH. Mathematical modelling of leprosy and its control. Adv Parasitol. 2015;87:33–51. 10.1016/bs.apar.2014.12.002 25765193

[15] Meima A, Gupte M, van Oortmarssen GJ, Habbema JDF. SIMLEP: a simulation model for leprosy transmission and control. Int J Lepr other Mycobact Dis. 1999;67(3):215–36. 10575401

[16] Fine PEM. Leprosy: The epidemiology of a slow bacterium. Epidemiol Rev. 1982;4(1):161–88. 10.1093/oxfordjournals.epirev.a036245 6754406

[17] Becx-Bleumink M. Relapses smong leprosy patients treated with multidrug therapy: experience in the leprosy control program of the All Africa Leprosy and Rehabilitation Training Center (ALERT) in Ethiopia; practical difficulties with diagnosing relapses; operational procedures and criteria for diagnosing relapses’. Int J Lepr. 1992;60(3):421–35. 1474281

[18] Schuring RP, Geluk A, Pahan D, Richardus JH, Oskam L. Protective effect of the combination BCG vaccination and rifampicin prophylaxis in leprosy prevention. Vaccin. 2009;27(50):7125–8. 10.1016/j.vaccine.2009.09.054 19786134

[19] Steinmann P, Cavaliero A, Aerts A, Anand S, Arif M, Sao SA, et al. The Leprosy Post-Exposure Prophylaxis (LPEP) programme: update and interim analysis. Lepr Rev. 2018;89:102–16. 10.47276/lr.89.2.102 PMC10174212 37180343

[20] World Health Organization. Global leprosy update, 2016: accelerating reduction of disease burden. Wkly Epidemiol Rec. 2017;92(35):501–20. Available from: http://apps.who.int/iris/bitstream/10665/258841/1/WER9235.pdf?ua=1 28861986

[21] Fine PEM, Sterne JAC, Ponnighaus JM, Bliss L, Saul J, Chihana A, et al. Household and dwelling contact as risk factors for leprosy in Northern Malawi. Am J Epidemiol Copyr O. 1997;146(1):91–102. 10.1093/oxfordjournals.aje.a009195 9215227

[22] World Health Organization. The END TB strategy: global strategy and targets for tuberculosis prevention, care and control after 2015. Geneva World Heal Organ. 2015;1:1–24. Available from: http://www.who.int/tb/post2015_TBstrategy.pdf

[23] Richardus JH, Tiwari A, Barth-Jaeggi T, et al. Leprosy post-exposure prophylaxis with single-dose rifampicin (LPEP): an international feasibility programme. Lancet Glob Health. 2021;9(1):e81–e90. 10.1016/S2214-109X(20)30396-X 33129378

[24] Tiwari A, Blok DJ, Arif M, Richardus JH. Leprosy post-exposure prophylaxis in the Indian health system: A cost-effectiveness analysis. PLoS Negelected Trop Dis. 2020;14(8):e0008521. 10.1371/journal.pntd.0008521 32750059 PMC7428216

[25] Tiwari A, Blok DJ, Suryawanshi P, Raikwar A, Arif M, Richardus JH. Leprosy services in primary health care in India: comparative economic cost analysis of two public-health settings. Trop Med Int Heal. 2019;24(2):155–65. 10.1111/tmi.13182 30444947 PMC7379621

[26] Mieras L, Anthony R, van Brakel W, Bratschi MW, van den Broek J, Cambau E, et al. Negligible risk of inducing resistance in Mycobacterium tuberculosis with single-dose rifampicin as post-exposure prophylaxis for leprosy. Infect Dis Poverty. 2016;5(1):46. 10.1186/s40249-016-0140-y 27268059 PMC4897814

[27] Cambau E, Saunderson P, Matsuoka M, et al. Antimicrobial resistance in leprosy: results of the first prospective open survey conducted by a WHO surveillance network for the period 2009–15. Clin Microbiol Infect. 2018;24(12):1305–10. 10.1016/j.cmi.2018.02.022 29496597 PMC6286419

[28] Sterling TR, Njie G, Zenner D, et al. Guidelines for the Treatment of Latent Tuberculosis Infection: Recommendations from the National Tuberculosis Controllers Association and CDC, 2020. MMWR Recomm Rep. 2020;69(1):1–11. 10.15585/mmwr.rr6901a1 32053584 PMC7041302

[29] Hooper PJ, Chu BK, Mikhailov A, Ottesen EA, Bradley M. Assessing progress in reducing the at-risk population after 13 years of the global programme to eliminate lymphatic filiriasis. PLoS Negl Trop Dis. 2014;8(11):e3333. 10.1371/journal.pntd.0003333 25411843 PMC4239000

[30] Joyce MP. Historic aspects of human susceptibility to leprosy and the risk of conjugal transmission. Mem Inst Oswaldo Cruz. 2012;107(Suppl. 1):17–21. 10.1590/s0074-02762012000900004 23283448

[31] Lockwood DNJ, Shetty V, Penna GO. Hazards of setting targets to eliminate disease: lessons from the leprosy elimination campaign. BMJ. 2014;348:1–5. 10.1136/bmj.g1136 24508610

[32] Blok DJ, de Vlas SJ, Richardus JH. Finding undiagnosed leprosy cases. Lancet Infect Dis. 2016;16(10):1113. 10.1016/S1473-3099(16)30370-X 27676349

[33] Feenstra SG, Nahar Q, Pahan D, Oskam L, Richardus JH. Recent food shortage is associated with leprosy disease in Bangladesh: a case-control study. PLoS Negl Trop Dis. 2011;5(5):e1029. 10.1371/journal.pntd.0001029 21572979 PMC3091833

[34] Kerr-Pontes LRS, Barreto ML, Evangelista CMN, Rodrigues LC, Heukelbach J, Feldmeier H. Socioeconomic, environmental, and behavioural risk factors for leprosy in North-east Brazil: results of a case–control study. Int J Epidemiol. 2006;35(4):994–1000. 10.1093/ije/dyl072 16645029

