# Supplementary material for: Number of people requiring post-exposure prophylaxis to end leprosy: A modeling study
Source: PLoS Negl Trop Dis. 2021 Feb 25;15(2):e0009146. doi: 10.1371/journal.pntd.0009146 (PMC7906365; doi:10.1371/journal.pntd.0009146)
Supplement: S1 Supporting Information File — Text document containing 1) quantification and assumptions of leprosy transmission parameters (Table A), 2) demographic data used (Table B), 3) quantification of household movement parameters (Table C), 4) epidemiologic data used (Table D), 5) overview of countries’ characteristics (Table E), 6) predicted impact of PEP on the leprosy new case detection rate in seven settings (Fig A), 7) predicted number of people requiring PEP per 100,000 in seven leprosy settings (Fig B), and 8) method to calculate country specific number of individuals requiring PEP (Equation A). (PDF) [file pntd.0009146.s001.pdf]

Supporting Information File 1

Contents

Table A. Quantification of leprosy transmission parameters in the model 2

Table B. Demographic data used to quantify the model. 4

Table C. Quantification of household movement parameters in the model 5

Table D. Epidemiologic data used to quantify the model 6

Table E. Overview of countries' characteristics 7

Fig A. Predicted impact of post-exposure prophylaxis (PEP) on the leprosy new case detection rate in seven settings. 10

Fig B. Predicted number of people requiring post-exposure prophylaxis (PEP) per 100,000 in seven leprosy settings. 11

Equation A. Calculation of country specific number of individuals requiring post-exposure prophylaxis (PEP) 12

References 13

Table A. Quantification of leprosy transmission parameters in the model

Parameters	Value		Source
**Natural history of infection**			
Proportion susceptible	20%		Assumption [1]
MB / PB ratio	India and Chhattisgarh	48 / 52	NLEP[a] [2]
	Brazil and Para State	66 / 34	Programme data[b] [3]
	Indonesia and Madura	83 / 17	Programme data[b] [3]
	Northwest Bangladesh	20 / 80	DBLM register [1, 4]
PB subclinical duration mean	4.2 years; SD =1.9 (gamma distributed)		[1, 5]
PB self-healing rate	20% per year		[1, 6]
MB subclinical duration mean	11.1 years; SD = 5.0 (gamma distributed)		[1, 5]
**Treatment**			
MDT use	1990 onwards		
MDT relapse rate	0.001 per year<br>To MB: 90%<br>To PB: 10%		[7, 8]
**Transmission**			
Infectivity function			[7]
PB	0		
Asymptomatic MB	Linear from 0 to 1		
Symptomatic MB	1		
Contact rate			
General population	India	*0.97; 95% CI: 0.93-1.01*	Calibrated[a] [3]
	Chattisgarh, India	*1.68; 95% CI: 1.64-1.72*	
	Brazil	*0.37; 95% CI: 0.33-0.41*	
	Pará State, Brazil	*0.54; 95% CI: 0.50-0.58*	
	Indonesia	*0.10; 95% CI: 0.06-0.15*	

	Madura, Indonesia	*0.24; 95% CI: 0.19-0.28*	
	Northwest Bangladesh	*1.33*	Calibrated[a] [1]
Within household	0.98		Calibrated[a] [1]
**Control**			
Passive case detection delays	India and Chattisgarh	2 years	Assumption informed by literature [3]
	Brazil and Para State	3 years	Assumption informed by literature [3]
	Indonesia and Madura	2 years	Assumption informed by literature [3]
	Northwest Bangladesh	2 years	Assumption informed by literature [1]
Contact tracing	India and Chattisgarh	-	[9]
	Brazil and Para State	59% coverage	Report of Ministry of Health Brazil [10]
	Indonesia and Madura	12% coverage	Programme data[b] [3]
	Northwest Bangladesh	90% coverage	COLEP study [11]
BCG protective effect	60%		[12]

[a] Calibrated to match modelled leprosy new case detection rate trend to data. We performed a grid search to obtain optimal parameter values. The simulated new case detection rates (mean of 100 runs) were compared to the data by a log-likelihood function assuming a Poisson distribution. These likelihood ratios were fitted to a polynomial regression meta model to obtain the optimal parameter values.
[b] Data obtained from leprosy programme coordinator

Table B. Demographic data used to quantify the model.

Country	Data	Years	Source
India	Population size & growth	1901-2011	Census India [13, 14]
	Fraction married	2001, 2011	Census India [15]
	Survival rates	1990, 2000, 2012	WHO
	Age-specific fertility rates	1992, 1998, 2006, 2011, 2013	DHS[a]; Census India [15]
	Age distribution	2013	DHS[a]
	Distribution of household size	2006	DHS[a]
Brazil	Population size & growth	1872-2010	Census Brazil [16]
	Fraction married	1990, 2000, 2010	IBGE[b] [17-19]
	Survival rates	1991, 2000, 2010	IBGE[b] [17-19]
	Age-specific fertility rates	1991, 1995, 2000, 2005, 2010	IBGE[b] [17-19]
	Age distribution	2010	IBGE[b] [19]
	Distribution of household size	2000	IBGE[b] [18]
Indonesia	Population size & growth	1850-2010	Census Indonesia (BPS) [20]
	Fraction married	1993, 1997, 2000, 2007	IFLS[c]
	Survival rates	1990, 2000, 2009	WHO
	Age-specific fertility rates	1971, 1980, 1990, 1991, 1997, 2003, 2007, 2012	DHS[a] [21, 22]
	Age distribution	2012	DHS[a] [22]
	Distribution of household size	2007	DHS[a] [21]
Bangladesh	Population size & growth	1800-2000	[23]; Bangladesh Bureau of Statistics [1]
	Fraction married	1961, 1974, 1981, 1991	Bangladesh Bureau of Statistics
	Survival rates	1966, 1971, 1987, 1993, 1994, 2000	Bangladesh Bureau of Statistics
	Age-specific fertility rates	1961, 1965, 1968, 1980, 1985, 1990, 1995	Bangladesh Bureau of Statistics
	Age distribution	2000	Bangladesh Bureau of Statistics
	Distribution of household size	2006	[24]

[a] Demography and Household Survey

[b] Brazilian Institute of Geography and Statistics

[c] Indonesian Family Life Survey

Table C. Quantification of household movement parameters in the model

Parameter	Value		Source
Fraction random movement	India and Chhattisgarh	0.98	Calibrated [a] [3]
	Brazil and Para State	0.75	Calibrated [a] [3]
	Indonesia and Madura	1.0	Calibrated [a] [3]
	Northwest Bangladesh	0.3	Calibrated [a] [1]
Fraction creates own household	India and Chhattisgarh	0.0	Calibrated [a] [3]
	Brazil and Para State	0.01	Calibrated [a] [3]
	Indonesia and Madura	0.0	Calibrated [a] [3]
	Northwest Bangladesh	0.2	Calibrated [a] [1]
Household size to move to	India and Chhattisgarh	Start =0, End = 4, Max =3 (Triangular distributed)	Calibrated [a] [3]
	Brazil and Para State	Start =0, End = 4, Max =2 (Triangular distributed)	Calibrated [a] [3]
	Indonesia and Madura	Start =0, End = 4, Max =2 (Triangular distributed)	Calibrated [a] [3]
	Northwest Bangladesh	Start =0, End = 50, Max =4 (Triangular distributed)	Calibrated [a] [1]
Fraction of married couple creating own household	0.25		Assumption [1]
Time until splitting of a married household from parental household	Mean = 12 (Exponentially distributed)		Assumption [1]
Fraction single widow(er)s moving back to children	India and Chhattisgarh	1.0	Calibrated [a] [3]
	Brazil and Para State	1.0	Calibrated [a] [3]
	Indonesia and Madura	1.0	Calibrated [a] [3]
	Northwest Bangladesh	0.0	Assumption [1]

[a] Calibrated such that the simulated distribution of household size matched the observed distribution. A grid search was conducted to find the optimal values. Goodness of fit of the distribution of household size was evaluated by a Chi-square test (See Blok et al.2015 and Fischer et al. 2010 for calibration results). We assumed that household structures in at the regional level in India, Brazil and Indonesia were similar to the household structures of the whole country.

Table D. Epidemiologic data used to quantify the model

Country	Data	Level	Years	Source
India	New case detection rate	Country	1991-2015	NLEP[a] [2]
		State: Chhattisgarh	2008-2015	
	MB proportion	Country	2011-2013	NLEP[a] [2]
	BCG coverage	Country	1980-2013	WHO [25]
Brazil	New case detection rate	Country	1990-2014	SINAN[b] [26]
		State: Pará	1990-2014	
	MB proportion	Country	2011-2013	SINAN[b] [26]
	BCG coverage	Country	1980-2013	WHO [25]
Indonesia	New case detection rate	Country	2000-2013	Program data[c] [3]
		District: Madura	2001-2010	
	MB proportion	Country	2000-2013	Program data[c] [3]
	BCG coverage	Country	1980-2013	WHO [25]
Bangladesh	New case detection rate	District: Northwest Bangladesh	2003	DBLM register [1]; [4]
	MB proportion	Country	2000-2013	DBLM register [1]; [4]
	BCG coverage	Country	1974-2000	Bangladesh reported [1]

[a] National Leprosy Elimination Programme;

[b] Sistema de Informações de Agravos de Notificação;

[c] Data obtained from leprosy program coordinator

Table E. Overview of countries' characteristics

Group	Country	District	Population size [27]	NCDR (per 100,000) [28]	MB/PB [28]	Assigned setting
1	India [29]	High endemic states [a]	743,217,770	14.4	51/49 [g]	A
		Low endemic states [b]	589,413,965	4.8	51/49 [g]	E
	Brazil [30]	High endemic states [c]	82,938,662	34.7	68/32 [g]	B
		Low endemic states [d]	119,829,300	6.1	68/32 [g]	F
	Indonesia [31]	High endemic districts [e]	98,817,757	14.8	85/15 [g]	G
		Low endemic districts [f]	138,823,569	1.6	85/15 [g]	C
2	DR Congo		81,339,988	4.8	61/39	G
	Ethiopia		104,957,438	3.6	83/17	G
	Nepal		29,304,998	10.5	55/45	E
	Bangladesh		162,951,560	25	20/80	D
	Myanmar		53,370,609	4.9	77/27	G
	Tanzania		57,310,019	3.7	88/12	G
	Sri Lanka		21,444,000	9.4	54/46	E
	Madagascar		25,570,895	7.2	85/15	G
	Philippines		104,918,090	1.7	88/17	G
	Nigeria		190,886,311	0.7	97/3	G
	Mozambique		29,668,834	4.5	82/18	G
	Ivory Coast		24,294,750	3.8	69/31	F
	South Sudan		12,575,714	6	89/11	G
	Egypt		97,553,151	0.7	89/11	G
	Sudan		40,533,330	1.6	87/13	G
	Angola		29,784,193	2.1	90/10	G
	Comoros		813,912	39	49/51	A
	Kiribati		116,398	190.6	27/73	D
	Micronesia		105,544	161	44/56	A
3	Tuvalu [h]		11,192	63.1	57/43	B
	Marshall Islands		53,127	150.8	71/29	C
	Suriname		563,402	4.5	48/52	E
	Trinidad and Tobago		1,369,125	1.7	57/43	E
	Yemen		28,250,420	1.3	53/47	E
	Eq· Guinea		1,267,689	0.8	50/50	E
	Vanuatu [h]		276,244	0.7	50/50	E
	Chad		14,899,994	0.6	40/60	E
	Costa Rica		4,905,769	0.3	56/44	E
	China, Macao SAR		622,567	0.3	50/50	E
	Singapore [h]		5,612,253	0.12	43/57	E
	USA		325,719,178	0.05	58/42	E
	Panama [h]		4,098,587	0.05	50/50	E
	Rwanda		12,208,407	0.3	37/63	E
	Bahrain [h]		1,492,584	0.4	50/50	E
	Honduras [h]		9,265,067	0.05	25/75	E
	Nauru [h]		13,649	16.03	50/50	E
	Oman [h]		4,636,262	0.1	25/75	E
	Liberia		4,731,906	8.1	54/46	E
	Guatemala [h]		16,913,503	0.02	33/67	E

Cape Verde [h]	546,388	0.8	50/50	E
American Samoa [h]	55,641	12.6	100/0	F
Palau [h]	21,729	9.3	100/0	F
Central Africa Rep	4,659,080	8.4	97/3	F
Solomon Islands	611,343	7.2	70/30	F
Guyana	777,859	6.7	73/27	F
French Guyana	275,713	4.7	77/23	F
Senegal	15,850,567	2.2	61/39	F
Maldives [h]	436,330	1.4	67/33	F
Guinea Bissau	1,861,283	1.2	64/36	F
Cameroon	24,053,727	1.2	72/28	F
Dominican Rep	10,766,998	1.2	71/29	F
Togo	7,797,694	1.1	68/32	F
Cambodia	16,005,373	1	69/31	F
Malaysia	31,624,264	0.7	70/30	F
Bolivia	11,051,600	0.6	62/38	F
Pakistan	197,015,955	0.2	72/28	F
Afghanistan	35,530,081	0.14	69/31	F
Samoa [h]	196,440	5.6	91/9	G
Paraguay	6,811,297	5.1	89/11	G
Timor-Leste	1,296,311	8.7	91/9	G
Somalia	14,742,523	4.4	88/12	G
Papua New Guinea	8,251,162	4.4	76/24	G
Burundi	10,864,245	4.1	78/22	G
Northern Mariana Islands [h]	55,144	3.6	100/0	G
Guinea	12,717,176	2.2	82/18	G
Sierra Leone	7,557,212	1.9	86/14	G
Niger	21,477,348	1.7	81/19	G
Cuba	11,484,636	1.6	87/13	G
Malawi	18,622,104	1.5	83/17	G
Congo	5,260,750	1.3	93/7	G
Bhutan	807,610	1.3	90/10	G
Burkina Faso	19,193,382	1.1	88/12	G
New Caledonia [h]	280,460	1.1	100/0	G
Ghana	28,833,629	1	97/3	G
Venezuela	31,977,065	1	81/19	G
Lao People's Dem Rep	6,858,160	0.9	78/22	G
Gabon	2,025,137	0.9	100/0	G
Argentina	44,271,041	0.7	86/14	G
Colombia	49,065,615	0.6	81/19	G
Uganda	42,862,958	0.5	86/14	G
Fiji [h]	905,502	0.3	100/0	G
Thailand	69,037,513	0.2	76/24	G
Jamaica [h]	2,890,299	0.2	100/0	G
Vietnam	95,540,800	0.15	83/17	G
Mexico	129,163,276	0.12	76/24	G
Ecuador	16,624,858	0.11	100/0	G

	Lesotho [h]	2,233,339	0.09	100/0	G
	Uruguay [h]	3,456,750	0.09	100/0	G
	Peru	32,165,485	0.07	100/0	G
	Morocco	35,739,580	0.07	80/20	G
	China	1,386,395,000	0.05	89/11	G
	Nicaragua [h]	6,217,581	0.02	100/0	G
	El Salvador [h]	6,377,853	0.02	100/0	G
	Rep of Korea [h]	51,466,201	0.01	75/25	G
	Sao Tome and Principe [h]	204,327	1.1	100/0	G
	Mali	18,541,980	1.3	87/13	G
	Benin	11,175,692	1.7	82/18	G
	Djibouti	956,985	1.3	100/0	G
	Gambia	2,100,568	0.6	92/8	G
	Iran	81,162,788	0.0	94/6	G
	Iraq [h]	38,274,618	0.0	100/0	G
	Kenya	49,699,862	0.2	89/11	G
	Saint Lucia [h]	178,844	5.0	89/11	G
	Saudi Arabia [h]	32,938,213	0.01	100/0	G

[a] India high endemic states: *Bihar, Chandigarh, Chhattisgarh, D & N Haveli, Gujarat, Jharkhand, Lakshadweep, Maharashtra, Odisha, Uttar Pradesh, West Bengal*

[b] India low endemic states: *A & N Islands, Andhra Pradesh, Arunachal Pradesh, Assam, Daman & Diu, Delhi, Goa, Haryana, Himachal Pradesh, Jammu & Kashmir, Karnataka, Kerala, Madhya Pradesh, Manipur, Meghalaya, Mizoram, Nagaland, Puducherry, Punjab, Rajasthan, Sikkim, Tamil Nadu, Telangana, Tripura, Uttarakhand*

[c] Brazil high endemic states: *Acre, Amapá, Amazonas, Bahia, Ceará, Espírito Santo, Goiás, Maranhão, Mato Grosso, Mato Grosso do Sul, Pará, Paraíba, Pernambuco, Piauí, Rondônia, Roraima, Sergipe, Tocantins*

[d] Brazil low endemic states: *Alagoas, Distrito Federal, Minas Gerais, Paraná, Rio de Janeiro, Rio Grande do Norte, Rio Grande do Sul, Santa Catarina, São Paulo*

[e] Indonesia high endemic districts: *Ahec, Banten, Gorontalo, Jawa Barat, Jawa Tengah, Jawa Timur, Maluku Utara, Nusa Tenggara Barat, Papua, Papua Barat, Sulawesi Selatan, Sulawesi Tengah, Sulawesi Tenggara, Sulawesi Utara, Sumatera Selatan*

[f] Indonesia low endemic districts: *Bali, Bengkuku, DI Yogyakarta, DKI Jakarta, Jambi, Kalimantan Barat, Kalimantan Selatan, Kalimantan Tengah, Kalimantan Timur, Kep· Bangka Belitung, Kepulauan Riau, Lampung, Maluku, Nusa Tenggara Timur, Riau, Sulawesi Barat, Sumatera Barat, Sumatera Utara*

[g] Country level figure

[h] Countries with less than 10 reported cases

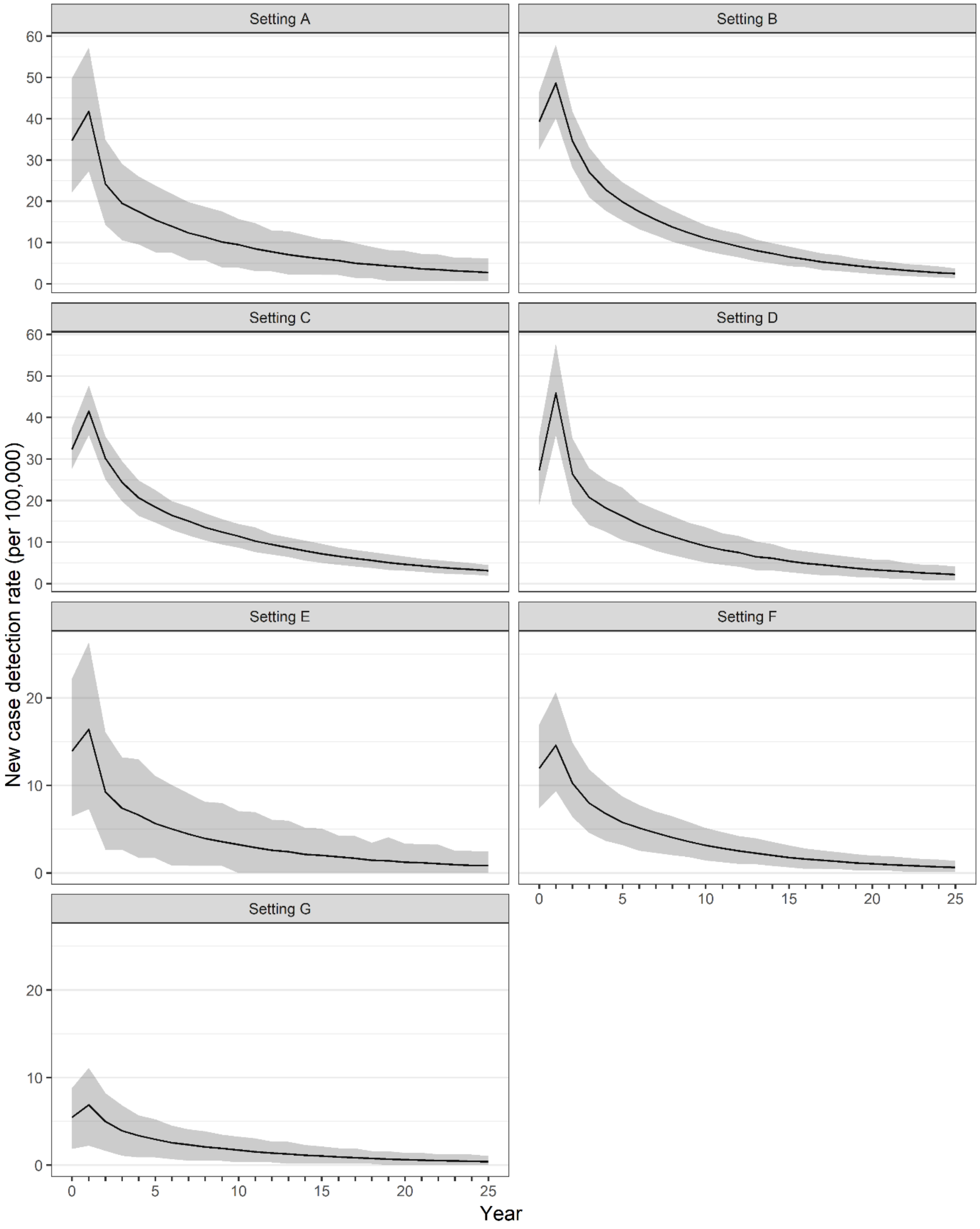

Fig A. Predicted impact of post-exposure prophylaxis (PEP) on the leprosy new case detection rate in seven settings.

The solid line represents the mean new cases detection rate. The shaded area represents the 95% uncertainty interval. Setting A is based on Chhattisgarh in India, setting B on Pará State in Brazil, setting C on Madura in Indonesia, setting D on Nilphamari and Rangpur in Bangladesh, setting E on India country-level, setting F on Brazil country-level, and setting G on Indonesia country-level.

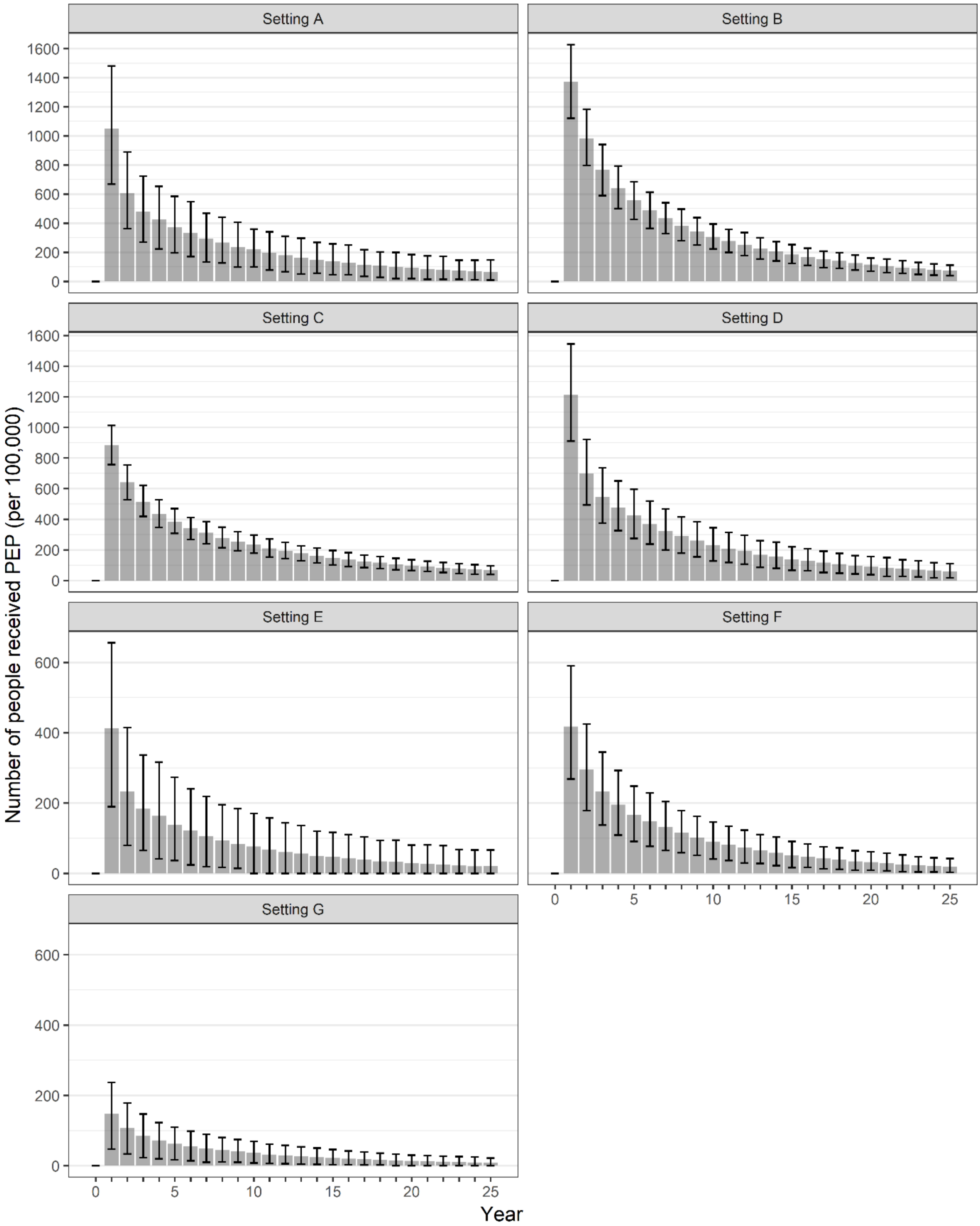

Fig B. Predicted number of people requiring post-exposure prophylaxis (PEP) per 100,000 in seven leprosy settings.

The bars represent the mean number of people requiring PEP. The error bars represent the 95% uncertainty interval. Setting A is based on Chhattisgarh in India, setting B on Pará State in Brazil, setting C on Madura in Indonesia, setting D on Nilphamari and Rangpur in Bangladesh, setting E on India country-level, setting F on Brazil country-level, and setting G on Indonesia country-level.

Equation A. Calculation of country specific number of individuals requiring post-exposure prophylaxis (PEP).

The number of new cases and the number of individuals requiring PEP per country are determined by the observed population size and the modelled new case detection rate (NCDR) and PEP rate (See equation (1) and (2).

Since the observed NCDR in a country may differ from the modelled NCDR of the assigned setting, we included a correction factor for the modelled NCDR: $NCDR_c^{obs}/NCDR_s^{sim}$

$Number\ of\ new\ cases\ detected_{c,s} = Population_c^{obs} \cdot \left(\frac{NCDR_s^{sim}}{100{,}000}\right) \cdot \left(\frac{NCDR_c^{obs}}{NCDR_s^{sim}}\right)$	(1)
$Number\ requiring\ PEP_{c,s} = Population_c^{obs} \cdot \left(\frac{PEP\ rate_s^{sim}}{100{,}000}\right) \cdot \left(\frac{NCDR_c^{obs}}{NCDR_s^{sim}}\right)$	(2)

with:

$Population_c^{obs}$:	Observed population size in country/state *c* (See Table B)
$NCDR_s^{sim}$:	Modelled new case detection rate per 100,000 population in setting *s* (See Fig A)
$NCDR_c^{obs}$:	Observed new case detection rate per 100,000 population in country/state *c* (See Table D)
$PEP\ rate_s^{sim}$:	Modelled number of individuals requiring PEP per 100,000 population in setting *s* (See Fig B)

References

1. Fischer EAJ, et al., Different mechanisms for heterogeneity in leprosy susceptibility can explain disease clustering within households. PLoS One. 2010;5(11):e14061.
2. Programme, NNLE, NLEP - Progress report for the year 2013-14. 2014, Central Leprosy Division: New Delhi.
3. Blok DJ, De Vlas SJ, and Richardus JH. Global elimination of leprosy by 2020: are we on track? Parasit Vectors. 2015;8:548.
4. Moet FJ, et al., Physical distance, genetic relationship, age, and leprosy classification are independent risk factors for leprosy in contacts of patients with leprosy. J Infect Dis. 2006;193(3):46-53.
5. Fine PE. Leprosy: the epidemiology of a slow bacterium. Epidemiol Review. 1982;4:161-188.
6. Sirumban P, Kumar A and Neelan PN. Healing time in untreated paucibacillary leprosy: a cross-sectional study. Int J Lepr Other Mycobact Dis. 1988;56(2):223-227.
7. Meima A, et al. The future incidence of leprosy: a scenario analysis. Bulletin of the World Health Organization. 2004;82(5):373-380.
8. Becxbleumink M. Relapses among leprosy patients treated with multidrug therapy - Experience in the leprosy control program of the All Africa Leprosy and Rehabilitation Training-Center (ALERT) in Ethiopia - practical difficulties with diagnosing relapses - operational procedures and criteria for diagnosing relapses. Int J Lepr Other Mycobact Dis. 1992;60(3):421-435.
9. Lockwood DN, Shetty V, and Penna GO. Hazards of setting targets to eliminate disease: lessons from the leprosy elimination campaign. BMJ. 2014;348:g1136.
10. Ministry of Health, National Hansen's Disease Control Programme report 2009-2010. 2011. Ministry of Health.
11. Moet FJ, et al. Effectiveness of single dose rifampicin in preventing leprosy in close contacts of patients with newly diagnosed leprosy: cluster randomised controlled trial. BMJ. 2008;336(7647):761-764.
12. Schuring RP, et al. Protective effect of the combination BCG vaccination and rifampicin prophylaxis in leprosy prevention. Vaccine. 2009;27(50):7125-7128.
13. Census India. Variation in Population since 1901. [cited 2015; Available from: http://censusindia.gov.in/Census_Data_2001/India_at_glance/variation.aspx.
14. Census India. Tabulations Plan of Census Year - 2011. [cited 2015; Available from: http://www.censusindia.gov.in/DigitalLibrary/TablesSeries2001.aspx.
15. Census India. Sample Registration System. [cited 2015; Available from: http://www.censusindia.gov.in/2011-Common/Sample_Registration_System.html.
16. IBGE. Censuses Historical Data. 2010 [cited 2015; Available from: https://ww2.ibge.gov.br/english/estatistica/populacao/censohistorico/default.shtm.
17. IBGE. Censo Demográfico 1991. 1991 [cited 2019 May 23]; Available from: https://biblioteca.ibge.gov.br/index.php.
18. IBGE. Censo Demográfico 2000. 2000 [cited 2019 May 23]; Available from: ftp://ftp.ibge.gov.br/Censos/Censo_Demografico_2000.
19. IBGE. Censo Demográfico 2010. 2010 [cited 2019 May 23]; Available from: http://www.ibge.gov.br/home/estatistica/populacao/censo2010.
20. BPS Statistics Indonesia. Population of Indonesia by Province 1971, 1980, 1990, 1995 , 2000 and 2010. [cited 2015; Available from: https://www.bps.go.id/statictable/2009/02/20/1267/penduduk-indonesia-menurut-provinsi-1971-1980-1990-1995-2000-dan-2010.html.
21. DHS, Indonesia Demographic and Health Survey 2007. 2007, BPS and Macro International: Calverton, Maryland, USA.
22. DHS, Indonesia Demographic and Health Survey 2012. 2012, BPS and Macro International: Calverton, Maryland, USA.

23. Gupta AD and Roy, SG. Population estimates for Bangladesh: the use of a specific transitional population model. Population Studies. 1976;30(1):15-33.
24. Fischer EAJ et al., The spatial distribution of leprosy in four villages in Bangladesh: an observational study. BMC Infectious Diseases. 2008;8.
25. World Health Organization. Reported estimates of BCG coverage. [cited 2015; Available from: http://apps.who.int/immunization_monitoring/globalsummary/timeseries/tscoveragebcg.html.
26. SINAN. Sistema de Informações de Agravos de Notificação. Available from: http://portalsaude.saude.gov.br/.
27. United Nations Population Division, World Population Prospects: 2019 Revision. Availble from: https://data·worldbank·org/indicator/sp·pop·totl
28. World Health Organization. Global leprosy update, 2016: accelerating reduction of disease burden. Wkly Epidemiol Rec. 2017;92:501–20. Available from: http://apps·who·int/iris/bitstream/10665/258841/1/WER9235·pdf?ua=1
29. National Leprosy Eradication Programme, Report for the year 2016-17, Central Leprosy Division, Government of India. Available from: http://nlep.nic.in/pdf/Annual%20report_%202016-17_rev.pdf
30. Leprosy Elimination Monitoring exercise in Brazil LEM-2012, Ministry of Health of Brazil (Brasilia - DF 2017)
31. Infodatin Kusta 2015, World Leprosy Day 2015, Data and Information Center of the Ministry of Health of Indonesia. Available from: http://www.depkes.go.id/resources/download/pusdatin/infodatin/infodatin_kusta.pdf
